# A prospective pilot study of two-session Gamma Knife surgery for large metastatic brain tumors

**DOI:** 10.1007/s11060-012-0882-8

**Published:** 2012-04-29

**Authors:** Shoji Yomo, Motohiro Hayashi, Claire Nicholson

**Affiliations:** 1 Saitama Gamma Knife Center, San-ai Hospital, 4-35-17 Tajima Sakura-ku, Saitama, 338-0837 Japan; 2 Department of Neurosurgery, Tokyo Women’s Medical University, Tokyo, Japan; 3 Regional Neurosciences Centre, Newcastle upon Tyne, UK

**Keywords:** Gamma Knife surgery, Stereotactic radiotherapy, Brain metastases

## Abstract

The purpose of this prospective study is to evaluate the efficacy and limitations of two-session Gamma Knife radiosurgery (GKS) alone for large metastatic brain tumors. Inclusion criteria were as follows: (i) patients with large metastatic brain tumors (volume >15 cm^3^ in the supratentorial region or >10 cm^3^ in the infratentorial region), and (ii) tumors not causing clinical signs of impending cerebral herniation. Twenty-eight lesions in 27 consecutive patients (18 men and 9 women, age range 32 to 88 years, median age 65 years) were included in this study. The radiosurgical protocol was as follows: 20–30 Gy given in two fractions 3–4 weeks apart. The local tumor control rate and the overall survival rate were calculated by using the Kaplan–Meier method. Median tumor volumes were 17.8 cm^3^ at first GKS and 9.7 cm^3^ at second GKS. Median follow-up time was 8.9 months. The local control rate was 85 % at 6 months and 61 % at 12 months. The overall survival rate after GKS was 63 % at 6 months and 45 % at 12 months. The 1-year rate of prevention of neurological death was maintained at 78 %. Mean Karnofsky performance status (KPS) improved from 61 [95 % confidence interval (CI), 57–71] at first GKS to 80 (95 % CI, 74–85) at second GKS; the best follow-up mean KPS was 85 (95 % CI, 78–91) (*p* < 0.001). Local tumor recurrence necessitated craniotomy in two patients and repeat GKS in three patients. Seventeen patients died, and the causes of death were as follows: 3 from local progression, 2 from meningeal carcinomatosis, and 12 from progression of the primary tumor. Delayed symptomatic perilesional edema developed in one patient and eventually resolved with conservative treatment. Two-session GKS for large brain metastases appears to be an effective treatment in terms of both local tumor control and neurological palliation with minimal treatment-related morbidity. These data suggest that two-session GKS could be used as an alternative to surgical resection of large tumors in patients with significant comorbidity and/or at an advanced age. The optimum regimen for dose and fraction schedule remains to be established.

## Introduction

Stereotactic radiosurgery (SRS) has become a treatment option of choice for the management of brain metastases [1–3]. Patients are generally considered candidates for SRS if their tumors are less than 10 cc in volume (<3 cm average diameter). Large-volume tumors have not hitherto been considered suitable for SRS because tumor size correlates with decreased response rate to radiation and increased risk of neurotoxicity [4, 5]. Standard treatment for large metastatic brain tumors is surgical resection, with adjuvant radiotherapy if feasible [5–8]. The number of patients with large brain metastases who are eligible for craniotomy is, however, fairly limited because of surgical accessibility of the tumor, the number of lesions, and the extent of systemic disease. Hypofractionated stereotactic radiation therapy (SRT) has been an alternative to improve the therapeutic ratio between tumor control and adverse radiation effects for treating large brain metastases [9–11]. Higuchi et al. [12] reported a new treatment method, where three-session stereotactic radiotherapy using Gamma Knife achieved excellent treatment results, presumably by virtue of significant tumor volume reduction during the interfraction intervals. The authors subsequently developed an alternative treatment paradigm comprising two-session GKS for large metastatic brain tumors, and the present study aims to evaluate the efficacy and limitations of such a treatment method.

## Materials and methods

### Patient characteristics

A prospective clinical trial was conducted to evaluate the efficacy and limitations of two-session GKS for large metastatic brain tumors. The institutional review board approved this prospective clinical trial in September 2009. Inclusion criteria were as follows: (i) patients with large metastatic brain tumors (volume > 15 cm^3^ in the supratentorial region or >10 cm^3^ in the infratentorial region), and (ii) tumors not causing clinical signs of impending cerebral herniation. The patients and/or their relatives were fully informed of the efficacy, invasiveness, and limitations of both radiosurgery and surgical resection; all gave written informed consent.

From October 2009 to April 2011, 30 consecutive lesions in 29 patients (20 men and 9 women) were enrolled in the present study, but 2 of these were excluded because the treatment protocol could not be completed (one due to acute lethal pancreatitis and the other to progression of systemic disease). Thus, 27 patients with 28 lesions were included in the study. The age range was from 32 to 88 years (median 65 years). In all cases, the diagnosis of the primary lesion had been confirmed histopathologically. Among patients harboring large metastatic tumors, six had undergone resective surgery before two-session GKS, three had had Ommaya reservoirs inserted, and two had already undergone whole-brain radiation therapy (WBRT) at their referring hospital.

### Radiosurgical techniques

GKS was performed using the Leksell G stereotactic frame (Elekta Instruments, Stockholm, Sweden). The frame was placed on the patient’s head under local anesthesia and with mild sedation. All patients underwent both stereotactic magnetic resonance (MR) imaging and computed tomography (CT). High-resolution three-dimensional (3-D) volumetric gadolinium-enhanced T1-weighted images and 2-mm-thick T2-weighted images were used for dose planning with Leksell Gamma Plan software (Elekta Instruments). The planning target volume was defined by adding no margin to the gross tumor volume. An isodose of less than 50 % was employed in most cases. At the end of the dose planning, spatial distortion of the MR scans was meticulously corrected for by checking CT images against MR images. The fraction protocol was as follows: 20–30 Gy in two fractions with 3–4 weeks between fractions. The interval between radiosurgical sessions was usually 3 weeks, but in some patients it was necessary to postpone the second procedure due to the schedule of concomitant systemic chemotherapy. The fractionated dose was calculated by using a linear quadratic formula, as described by Brenner et al. [13]. Assuming alpha/beta to be 10 for brain metastases, 20–30 Gy in two fractions was approximately equivalent to a single administration of 16–23 Gy. The Leksell Model C Gamma Knife was used in all cases. Concomitant small- to medium-sized metastases were also treated with SRS at a prescription dose ranging from 18 to 22 Gy (median 20 Gy) at either the first or the second session. Patient characteristics, tumor location, and treatment prior to two-session GKS are presented in Table 1.

**Table 1:** Patient characteristics

Characteristic	Value
Total no. of patients	27
Men/women	18/9
Age (years), median (range)	65 (32–88)
KPS, mean (range)	61 (30–90)
RTOG-RPA classification	
Class 1	0
Class 2	9
Class 3	18
No. of intracranial lesions, median (range)	2 (1–6)
Location	
Supratentorial	13
Infratentorial	15
Primary tumors	
Lung	17
Breast	4
Colon and rectum	4
Esophagus	1
Ovary	1
Procedures prior to GKS	
Craniotomy	6
Ommaya reservoir	3
WBRT	2

*RTOG* Radiation Therapy Oncology Group, *RPA* recursive partitioning analysis, *GKS* Gamma Knife surgery, *WBRT* whole-brain radiation therapy

### Post-GKS management and follow-up evaluation

In patients with significant neurological symptoms, administration of oral steroids (usually dexamethasone 2 mg per day) was continued between the two sessions and was tapered off and discontinued over a maximum of 4 weeks after the second session. Clinical follow-up comprised neuroradiological evaluation of bimonthly MR images as well as neurological evaluation, including KPS, in order to provide early identification of local and distant tumor recurrences. The change in tumor volume was calculated from 3-D volumetric MR images. Local control failure was defined as an increase in target lesion volume of at least 20 % compared with the smallest documented tumor volume on MRI. Delayed radiation injury was cautiously differentiated from tumor recurrence using the T1/T2 mismatch method [14] and the signal–intensity time curve obtained from dynamic susceptibility-weighted contrast-enhanced perfusion MR imaging [15]. Additional GKS was possible in principle, provided the volume of local tumor recurrence was small enough for single-session SRS. Surgical removal was indicated when clinical signs of cerebral herniation developed, with a radiological diagnosis of local tumor progression or radiation necrosis. Any adverse events attributable to SRS were evaluated by National Cancer Institute Common Terminology Criteria for Adverse Events (CTCAE) version 4.03. Patients with symptomatic delayed radiation injury were treated with intensive oral steroids and hyperbaric oxygen therapy [16]. When metachronous brain metastases were shown as small-enhanced lesions in serial MR imaging, they were managed with additional SRS. Neurological death was defined as death attributable to intracranial metastases, including tumor recurrence and carcinomatous meningitis.

### Statistical analysis

The date of data analysis was October 21st 2011. The local tumor control rate and the overall survival rate were calculated by using the Kaplan–Meier method. The intervals from the date of the first intervention for brain metastases until the date of confirmed local control failure or the date of death were calculated. The rate of prevention of neurological death was similarly calculated with the interval from the date of the first GKS until the date of neurological death. Death due to extracranial progression was regarded as a “censoring” in the estimation of the rate of prevention of neurological death. In order to assess the impact of this treatment on the quality of life of patients, the KPSs at each clinical stage were compared by using the Friedman test. All statistical analyses were performed with commercially available statistics software (Prism, version 5.0; GraphPad, La Jolla, CA). A *p* value of <0.05 was considered statistically significant.

## Results

Median tumor volume was 17.8 cm^3^ (range 10.0–53.3 cm^3^). The median prescription dose at the tumor margin for the first intervention was 13.3 Gy (range 10–16 Gy), and the median marginal isodose was 42 % (range 40–50 %). Similarly in the second intervention, the median prescription dose at the tumor margin was 13.3 Gy (range 10–15 Gy) and the median marginal isodose was 44 % (range 40–60 %). Median tumor volume was 9.7 cm^3^ at the time of the second GKS (46 % volume reduction). With the exception of one case, the tumor volume was reduced at the second session compared with the original volume.

**Fig. 1:**
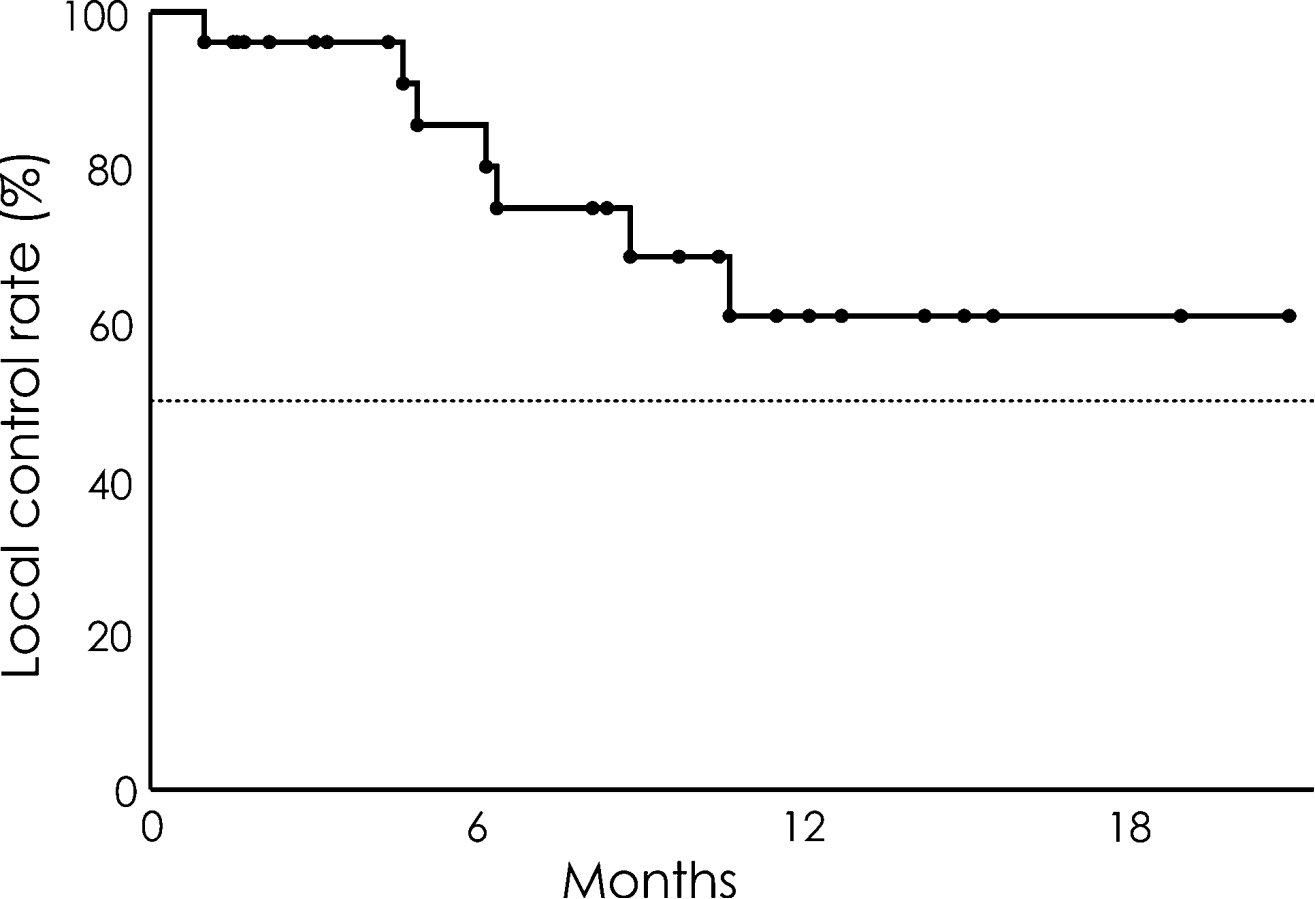
Local tumor control rate

**Fig. 2:**
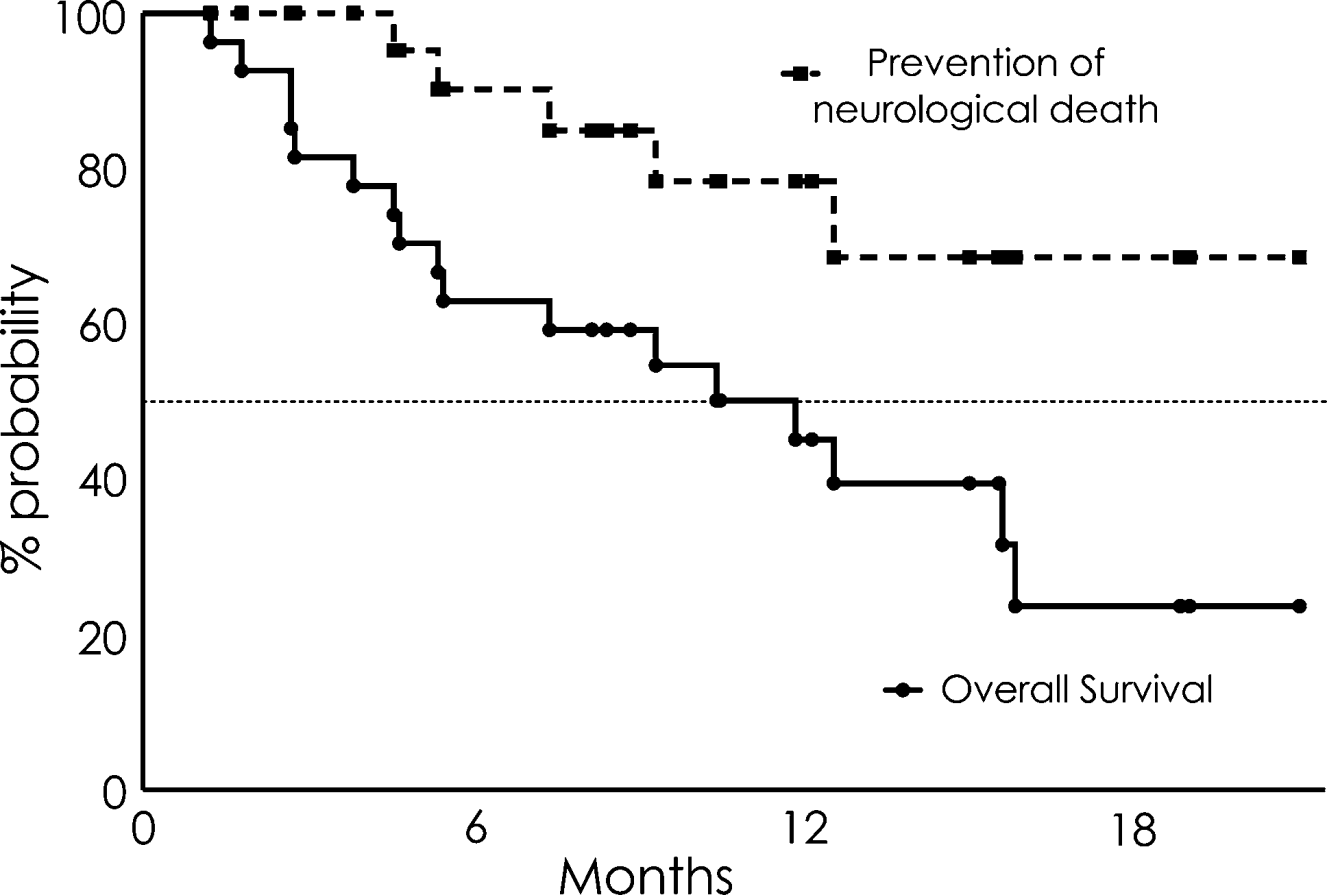
Overall survival rate and rate of prevention of neurological death

Median follow-up time was 8.9 months (range 1–21 months). Six patients (21 %) showed failure of local control between 1 and 13 months after two-session GKS (median 6.2 months). The local control rate was 85 % and 61 % at 6 and 12 months, respectively (Fig. 1). The overall survival rate after GKS was 63 and 45 % at 6 and 12 months, respectively. Median survival time was 11.9 months (95 % CI, 4.67–15.63 months) (Fig. 2). Similarly, the rate of prevention of neurological death after GKS was 90 and 78 % at 6 and 12 months, respectively (Fig. 2).

Follow-up neurological evaluation showed improvement of 20 or more points on the KPS in 17 patients (61 %), in terms of motor weakness, seizures, and higher brain functions. In the other cases (36 %), with the exception of one patient, the pre-existing neurological deficit remained stable. Mean KPS improved from 61 (95 % CI, 57–71) at first GKS to 80 (95 % CI, 74–85) at second GKS and 85 (95 % CI, 78–91) at best follow-up time (*p* < 0.001, Friedman test).

Seventeen patients died during follow-up, and the causes of death were as follows: 3 of intracranial local progression, 2 of meningeal carcinomatosis, and 12 of progression of the primary lesion. Subsequent interventions were needed in 13 cases. Salvage surgical resection was carried out in two patients because of increasing size of the metastasis. One patient underwent surgical resection 2 weeks after the second session because of progressive left hemiparesis; the resection specimen revealed tumor progression. His neurology was unchanged postoperatively. Another patient underwent craniotomy 5 months after two-session GKS, and histopathology confirmed predominantly radiation necrosis but with some viable tumor cells seen. Repeat GKS was performed for 4 local tumor recurrences in 3 patients, and for new distant metastases in 11 patients. Local control was achieved in all recurrent tumors treated with additional procedures.

**Fig. 3:**
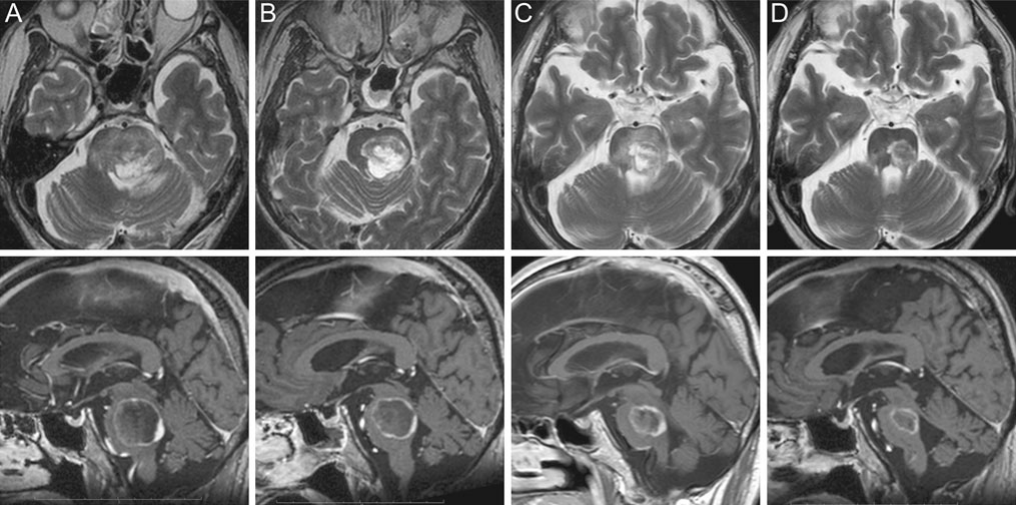
A 72-year-old man with small cell lung cancer presented with reduced conscious level. MR imaging demonstrated a large necrotic metastatic brain tumor in the pons. Due to the severity of neurological symptoms, the risk of WBRT was rated as high. As an alternative treatment option, the patient was allocated to two-session GKS. The first treatment delivered 10 Gy to the 40 % isodose (Fig. 3a). Three weeks later, at the second session, significant tumor volume reduction was observed and 10 Gy to the 40 % isodose was delivered to the tumor margin (Fig. 3b). Follow-up MR imaging after 4 months showed a considerable decrease in tumor size but brain stem perifocal edema (Fig. 3c). The perifocal edema subsided by the 8-month follow-up (Fig. 3d), and the KPS improved from 30 to 70. Although transient neurological deterioration occurred due to delayed radiation injury, the patient could lead an independent life until 2 months before he died from systemic disease progression

Two patients had transient emesis, both of whom required brief hospitalization for steroid administration (CTCAE grade 3 toxicity). These patients recovered to their preradiosurgical functional status within 1 week. In one patient, T2-weighted MR imaging 4 months after the first intervention demonstrated a high-intensity area in the surrounding brain stem, suggestive of delayed radiation toxicity, resulting in neurological deterioration, including hemiparesis (CTCAE grade 3 toxicity). These neurological and radiological changes eventually improved with oral steroids (Fig. 3). Treatment results are summarized in Table 2.

**Table 2:** Treatment results and outcome in 27 patients after two-session GKS for large brain metastases

Characteristic	Percentage points	No. of patients
1-Year local control rate	61	
1-Year overall survival rate	45	
1-Year rate of prevention of neurological death	78	
Mean KPS (95 % CI)		
At first GKS	61 (57–71)	
At second GKS	80 (74–85)	
Best in follow-up	85 (78–91)	
Local recurrence		5
Symptomatic radiation injury		1
Distant new metastases		11
Subsequent treatment		
Craniotomy for local recurrence		2
GKS for local recurrence		3
GKS for new metastases		11
Cause of death		
Systemic disease progression		12
CNS progression		5

*GKS* Gamma Knife surgery, *CI* confidence interval, *KPS* Karnofsky performance scale, *CNS* central nervous system

## Discussion

Surgical resection is a standard treatment option for large brain tumors. However, surgical resection for metastatic brain tumors in eloquent locations (primary motor cortex, thalamus, brain stem) carries significant neurological risk, even with modern neurosurgical techniques. Other factors such as patient age, comorbidity, and short life expectancy may make invasive treatment unattractive.

Single-session radiosurgery is suitable for small tumors, ideally those which are less than 3 cm in diameter or 10 cm^3^ in volume. Single-session radiosurgery for large tumors has the significant disadvantage of a narrow therapeutic ratio. To avoid radiation-induced complications for large lesions, the dose for a single irradiation may have to be limited to below the dose needed for effective tumor control; therefore, dose fractionation is a potential alternative strategy for increasing the total dose delivered to the lesion. Recent studies have found that hypofractionated SRT for brain metastases can achieve satisfactory tumor control [10, 17]. Higuchi et al. [12] developed a unique fraction schedule using Gamma Knife, which differed significantly from the schedules in the aforementioned studies, where favorable tumor control was achieved with three fractions separated by a 2-week interfraction interval. The advantage of their technique was that it allowed the radiation dose to normal brain tissue to be reduced for the second and third treatments owing to continual tumor volume reduction. In our series, using an interfraction time of 3–4 weeks, only one tumor was larger at the second intervention than at the first. From the viewpoint of radiation biology, however, a long interfraction time theoretically carries a potential concern about “repopulation” of tumor cell kinetics after radiation. The effects of the repair of sublethal damage to DNA on the efficacy of treatment should be taken into account, but parameters to quantify these effects have not yet been established [18]. The accumulation and analysis of clinical outcomes should allow us to determine the clinical effect of a long interfraction time on outcome.

The characteristic benefit of GKS in terms of its inherent steep radiation fall off can be maintained even in two-session procedures for larger tumors, thus protecting adjacent brain tissue from radiation-induced injury. Based on our own experience and in accordance with the literature, a marginal dose of 10-15 Gy per fraction is safe and effective. Moreover, our treatment technique includes using an intentionally low equivalent isodose at the margin of the tumor. This strategy means that a high average dose is delivered inside the target volume while minimizing the radiation dose to the “radiation penumbra,” which includes the surrounding brain tissue. Early significant tumor volume reduction with this approach could provide substantial neurological palliation with minimal invasiveness, even for patients with significant comorbidity and/or low performance status. GKS as two-session treatment seems to be associated with a low risk of complications and requires only a short period of hospitalization compared with both surgery and WBRT.

Local control of large metastatic lesions was not achieved even after two-session GKS in six patients (21 %). This number appears no better than in other previous studies, which may be attributed to a definition of local control failure different from in other series. The present study used the response evaluation criteria in solid tumors (RECIST) guidelines, because immediate salvage GKS was preferred if close clinical and imaging monitoring led to early detection of local recurrence or new lesions. The RECIST definition was much stricter than in most other studies, which usually define local recurrence as an increase in size of 25 % or more compared with pretreatment size [12, 19]. Even after carefully considering the difference of criteria used for evaluating local control failure, the resulting local tumor control in the present study was not the same as that of single-session SRS for small to medium-sized brain metastases. Tumor volume has been proved by many investigators to be a predictive factor for local tumor control after SRS [20–22], which would be one of the inevitable limitations of our treatment approach. Of six patients with local recurrence, three underwent salvage GKS procedures, resulting in successful tumor control in all cases. New intracranial metastases appeared after two-session GKS in 11 patients (41 %). These metachronous lesions were managed as described above. Thus, neurological death could be avoided in many patients by active continued management of intracranial metastases. There was, however, a limitation in this type of local treatment. It is difficult to control meningeal spreading of metastasis by using stereotactic irradiation alone. WBRT should be considered as a possible salvage treatment when carcinomatous meningitis develops.

Overall survival in this series was not found to be better than in other studies of SRS for brain metastases [12, 19, 23]. All of our patients were in RPA class II or III [24]. There were no RPA class I patients in this cohort. Given that prognosis is strongly related to RPA class, our treatment results are comparable to those of other series [24–26].

We had one complicated case with delayed radiation toxicity (Fig. 3). In this case, the signal changes on MR imaging were fortunately reversible and the patient eventually recovered to their pre-intervention neurological level, although their quality of life was significantly affected for a period of time by this complication. Even with this treatment technique, it appears to be difficult to avoid radiation side-effects completely when treating highly radiosensitive regions such as the brainstem.

One of the reasons behind the introduction of two-session treatment is related to economic constraints. The public health insurance system in our country will fund GKS as a single-session radiosurgical treatment but does not currently approve a two- or three-session treatment method. The second session has to be conducted free of charge. Consequently two-session GKS method delivers cost-effective medical care because this treatment costs approximately 20 % less than other LINAC-based fractionated SRT modalities in our domestic medical systems. Time constraints for patients with poor prognosis should also be taken into account. Two-session GKS takes less time than other radiotherapeutic modalities for brain metastasis, which is attractive and beneficial for patients with advanced cancer.

## Conclusions

Two-session Gamma Knife radiosurgical treatment for large brain metastases represents a safe treatment modality providing neurological palliation in the short to medium term, with acceptable tumor control rates and low morbidity. This treatment method can also be used for large tumors in eloquent locations either after WBRT or as the primary treatment for patients who are not suitable for surgical resection. The optimum regimen for dose and fraction schedule remains to be elucidated.
